# Outcomes in Nonagenarians with Hip Fractures Treated Conservatively and Surgically

**DOI:** 10.5704/MOJ.2111.004

**Published:** 2021-11

**Authors:** R Malhotra, SS Huq, M Chong, D Murphy, ZJ Daruwalla

**Affiliations:** Department of Orthopaedics, National University Hospital of Singapore, Singapore

**Keywords:** hip fracture, nonagenarians, mortality, morbidity, mobility

## Abstract

**Introduction:**

We aimed to assess the clinical outcomes in nonagenarians following a hip fracture. We also further investigated the factors that influence these outcomes, such as method of treatment (operative versus conservative), co-morbidities, and pre-morbid function.

**Materials and methods:**

We studied 65 nonagenarians that were identifiable from our hospital hip fracture database. We reviewed various parameters of these patients admitted after sustaining a hip fracture (neck of femur or intertrochanteric) and investigated how these parameters affected patient outcomes. The main outcomes studied were: inpatient morbidity, and mortality at one year.

**Results:**

Inpatient morbidity was more likely in patients with an ASA grade of 3 to 5. Urinary tract infection was the most common medical complication. The 1-year mortality was 15.4% and was significantly influenced by advancing age. Surgically managed patients had a 1-year mortality rate (14.3%) slightly less than non-operative patients (17.4%). Post injury mobility was significantly better in those who received operative treatment with 63% of surgical cases regaining ambulatory status versus 7% of conservatively managed patients.

**Conclusions:**

We presented the outcomes of hip fractures in an extreme age group in the population. In nonagenarians with hip fractures surgery was associated with a 1-year mortality rate of 14.3% which is comparable to the general hip fracture population and less than the mortality rate of conservatively managed patients (17.4%). The primary advantage of surgery would be that two-thirds of patients return to ambulatory status. This information is useful to counsel patients and their families especially since the elderly are often more fearful of surgical intervention.

## Introduction

Hip fractures are on the rise globally with the World Health Organization stating that, “approximately 1.66 million hip fractures occur each year worldwide” and, “the incidence is set to increase four-fold by 2050 because of the increasing number of older people^1^”. While Singapore’s total population could fall between 6.5 and 6.9 million by 20302, it is expected that 19% will be persons aged 65 years and above^3^. With increased life expectancy, it is anticipated that many in this group who sustain a hip fracture will be in their nineties. These nonagenarians may require particular attention because age has long been accepted to be a factor strongly associated with mortality^4^.

Conservative management of hip fractures may be considered for patients deemed to be at very high risk for surgery, otherwise surgery is the gold standard of care^5^. Even in nonagenarians, surgical management of hip fractures is common. In some populations conservative treatment is seen in only 1% of patients, or in 3% of nonagenarians^6^.

In Singapore, it would seem that the elderly frequently refuse to undergo recommended surgery. A recent Singaporean study showed that a third of patients with hip fractures opt for conservative treatment^7^, while another local study showed nearly half of nonagenarians with hip fractures are managed conservatively^8^.

We aimed to analyse key outcomes in nonagenarians who sustain a hip fracture. Our primary outcomes were to evaluate the inpatient morbidity and mortality in nonagenarians with hip fractures treated both conservatively as well as surgically. A secondary observation of mobility following injury was also assessed. The following factors were also analysed for their influence on these three outcomes: co-morbidities (hypertension, diabetes, dialysis dependence), laboratory investigations on admission (renal function and full blood count), and pre-injury mobility.

## Materials and Methods

All subjects in this study were retrieved from our institution’s database for hip fractures. Patient data was reviewed over a three-year period from 1st January 2010 to 31st December 2012 inclusive^9^. This study was approved by our Institutional Review Board. Inclusion criteria for this study were all hip fracture patients aged 90 to 99, regardless of whether they were admitted directly under an orthopaedic team or not. Patients with pathological fractures were excluded. Patients were all offered surgical management by the consultant in charge, but non-operative treatment ensued if patients or their family refused surgery after a complete discussion with the orthopaedic team.

The primary outcome measures were investigated. Firstly, the incidence of inpatient morbidity, and secondly the one-year mortality. We made attempts to evaluate mobility in patients suffering with hip fractures. Premorbid mobility could be scored the New Mobility Score (NMS) (Table I)^10^. This scores patients’ ability to mobilise within and outside of their home environment and the amount of assistance needed. The worst score was zero, with a maximal score of 9 points. Quantifying premorbid mobility is useful when using it as a predictive factor for other outcomes in statistical analysis. However, the post-injury mobility was simplified to two categories, ability to ambulate or not.

**Table I:** New mobility score (NMS)

Mobility	No difficulty	With aid	With help from another person
Able to get about the house	3	2	1
Able to get out of the house	3	2	1
Able to go shopping	3	2	1

Clinical records were searched to extract the admission and follow-up data. Demographics and in-patient morbidity data were available from hospital electronic records of the admission. Mobility data was assessed on follow-up visits and documentation of this was visible in the out-patient clinic notes as documented by doctors or therapists. The patients were considered immobile if they were bed bound or wheelchair bound and ambulatory if they were able to mobilise independently or with assistance from another person or walking device such as a stick or frame. For mortality data in patients lost to follow-up, we contacted patients or their families on telephone to enquire on their well-being or date of demise if applicable.

All data collected was inputted electronically on a data collection form which was stored on a password protected computer in the orthopaedic department research office.

Variables that were chosen for analysis included:

- Demographic data: age, sex and race
- Type of hip fracture
- Type of management performed (nature of operative or non-operative treatment)
- Pre-injury mobility was scored on the New Mobility Score (NMS)
- American Society of Anaesthesiologists (ASA) grade
- Lab results: haemoglobin, platelets, creatinine and urea (as measured on day of admission)
- Presence of the following co-morbidities: hypertension, diabetes and dialysis requirement.

Univariate analyses were performed for continuous data to identify predictive factors relating to mobility, morbidity and mortality. T-tests were used for normally distributed data and Mann-Whitney-U test for non-parametric data. Multivariate analyses were carried out using logistic regression analysis to further investigate factors predicting inpatient morbidity and 1-year mortality. Statistical analysis of the data was performed using SPSS Version 21.0 [IBM Corp 2012]^11^. All univariate tests were two-tailed. Statistical significance was set at p<0.05 for all analyses.

## Results

A total of 65 patients met the inclusion criteria for analysis in this study with a median age of 91 (range 90-99). Mean pre-injury NMS value was 4.0 with a standard deviation (SD) of 2.1. Fifty-eight (89.2%) subjects were female and 59 (90.8%) were of Chinese ethnicity. The total incidence of inpatient morbidity was 41.5% while one-year mortality was 15.4%. Surgical management was performed in 42 (64.6%) cases and median follow-up was 42 months (range 0-66). The descriptive data of all patients are summarised in Table II and Table III. Table IV lists the variables that were analysed as potential predictors.

**Table II:** Descriptive statistics for continuous variables

Variable	Average^a^	SD^b^/Range
Age	91^c^	90-99^d^
Haemoglobin	11.7	1.7
Platelets	224	69
Urea	6.6^c^	3.2-23.4^d^
Creatinine	89^c^	42-223^d^
Pre-morbid NMS^e^	4.0	2.1

^a^Expressed as mean unless stated; ^b^Standard Deviation; ^c^Median; ^d^Range; ^e^New Mobility Score

**Table III:** Descriptive statistics for categorical data

Variable	Frequency	%
Sex		
Female	58	89.2
Male	7	10.8
Race		
Chinese	59	90.8
Malay	2	3.1
Others	4	6.2
Side of Hip Fracture		
Left	42	64.6
Right	23	35.4
Type of Hip Fracture		
NOF^a^ Fracture	30	46.2
IT^b^ Fracture	35	53.8
ASAc Grade		
I	2	3.1
II	12	18.5
III	45	69.2
IV	6	9.2
V	0	0.0
Hypertension		
Yes	38	58.5
No	27	41.5
Diabetes Mellitus		
Yes	12	18.5
No	53	81.5
End-Stage Renal Failure		
Yes	0	0.0
No	65	100.0
Management Type		
Conservative	23	35.4
*NOF^a^ Fractures*	*11*	*16.9*
*IT^b^ Fractures*	*12*	*18.5*
*Surgical*	*42*	*64.6*
*NOF^a^ – Hemiarthroplasty*	*17*	*26.2*
*NOF^a^ – Cannulated Screws*	*2*	*3.1*
*NOF^a^ – Total Hip Arthroplasty*	*0*	*0.0*
*IT^b^ – DHS*	*14*	*21.5*
*IT^b^ – IM Nail*	*9*	*13.8*
Inpatient Morbidities		
Number of Patients Affected	27	41.5
*UTI^d^*	*11*	*16.9*
*Pneumonia*	*5*	*7.7*
*MI^e^*	*5*	*7.7*
*DVT^f^*	*4*	*6.2*
*PE^g^*	*2*	*3.1*
*Other Cardiac Causes*	*2*	*3.1*
*CVA^h^*	*2*	*3.1*
*Others*	*4*	*6.2*

^a^Neck of Femur; ^b^Intertrochanteric; ^c^American Association of Anaesthesiologists; ^d^Urinary Tract Infection; ^e^Myocardial Infarction; ^f^Deep Vein Thrombosis; ^g^Pulmonary Embolism; ^h^Cerebrovascular Accident

**Table IV:** Summary of all potential predictive factors

Category	Predictive Factors
Demographic	Age^a^
	Sex
	Race^b^
Fracture-related	Type of Fracture (NOF^c^ or IT^d^)
	Type of Management (Surgical or Conservative)
Medical Condition	ASA^e^ Grade (Grade I-II or Grade III-V)
	Hypertension
	Diabetes Mellitus
	End-Stage Renal Failure
	Inpatient Morbidity Acquired^f^
	Pre-Morbid NMS^g^
Biochemistry	Haemoglobin
	Platelets
	Urea^h^
	Creatinine^h^

^a^Logarithmic transformation unable to normalize data, therefore variable not used in linear regression analyses; ^b^Not analysed due to lack of subjects from non-Chinese ethnicities; ^c^Neck of Femur; ^d^Intertrochanteric; ^e^American Society of Anaesthesiologists; ^f^Not analysed due to lack of ESRF patients; ^j^Used in mortality analysis only; ^g^New Mobility Score; ^h^Data required logarithmic transformation to normalize data

Overall, 41.5% of patients had a least one medical complication during their admission. The most common causes of morbidity were urinary tract infection (16.9%), pneumonia (7.7%), ischaemic cardiac event (7.7%), deep vein thrombosis (6.2%), pulmonary embolism (3.1%) and stroke (3.1%) (Table II). Tables V summarise the univariate analyses in relation to predicting inpatient morbidity. Although no variables significantly affected morbidity, two variables approached statistical significance. Advancing age (p=0.056) and having a higher ASA grade of III or IV (OR 3.259; p=0.085) may increase the chances of having inpatient morbidity.

**Table V:** (a) Univariate analyses of potential continuous predictive variables for inpatient morbidity. (b) Univariate analyses of potential categorical predictive variables for inpatient morbidity (a)

Variable	Test Statistic^a^	95% CI^b^	p-value
Age	653.5^c^	-	0.056*
Pre-Morbid NMS^d^	0.282	-0.9-1.2^e^	0.779
Haemoglobin	0.532	-0.6-1.1^e^	0.597
Platelets	-1.624	-62-6^j^	0.109
Urea	519.0^c^	-	0.936
Creatinine	577.5^c^	-	0.390

*Approaching significance (p<0.1); ^a^Independent t-test statistic unless stated; ^b^Confidence Interval; ^c^Mann-Whitney-U statistic (U); ^d^New Mobility Score; e95% CI of mean difference

**Table:** (b)

Variable	Comparison Groups	n (%)	Odds Ratio	95% CI^a^	p-value
Sex	Male vs Female	7(11) vs 58 (89)	1.894	0.339-10.579	0.690^b^
Type of Fracture	NOF^c^ vs IT^d^	30 (46) vs 35 (54)	1.481	0.549-3.994	0.437
Surgical treatment	Yes vs No	23 (35) vs 42 (65)	1.131	0.404-3.166	0.814
ASA^e^ Grade	III-V vs I-II	51 (78) vs 14 (22)	3.259	0.812-13.085	0.085*
Hypertension	Yes vs No	38 (58) vs 27 (42)	1.376	0.502-3.776	0.535
Diabetes Mellitus	Yes vs No	12 (18) vs 53 (82)	1.006	0.282-3.588	1.000^b^

*Approaching significance (p<0.1); ^a^Confidence Interval; ^b^Fisher’s Exact Test; ^c^Neck of Femur; ^d^Intertrochanteric; ^e^American Society of Anaesthesiologists

However, when entering these variables into a multivariate binary logistic regression model, neither proved to have statistical significance. Nagelkerke’s R2 for this model was 0.126 implying that age and higher ASA grades accounted for 12.6% of the variability in predicting incidences of inpatient morbidity.

Out of 65 patients, 10 died within one year, giving a 1-year mortality of 15.4%. Comparing surgical and non-surgical patients, 6 out of 42 surgically managed patients died within one year (14.3%) compared to 4 out of 23 conservatively managed patients (17.4%). Tables VI show one year mortality data and suggests only older age to be a significant risk factor for this dependent variable (p=0.004) and surgery vs conservative was not significant.

**Table VI:** (a) Univariate analyses of potential continuous predictive variables for one year mortality. (b) Univariate analyses of potential categorical predictive variables for one year mortality (a)

Variable	Test Statistic^a^	95% CI^b^	p-value
Age	430.5^c^	-	0.004*
Pre-Morbid NMS^d^	-1.317	-1.8-0.4^e^	0.193
Haemoglobin	-1.500	-2.1-0.3^e^	0.139
Platelets	-1.499	-83-12^e^	0.139
Urea	363.5^c^	-	0.107
Creatinine	345.5^c^	-	0.200

*Statistically significant (p<0.05); ^a^Independent t-test statistic unless stated; ^b^Confidence Interval; ^c^Mann-Whitney-U statistic (U); ^d^New Mobility Score; ^e^95% CI of mean difference

**Table:** (b)

Variable	Comparison Groups	n (%)	Odds Ratio	95% CI^a^	p-value
Sex	Male vs Female	7(11) vs 58 (89)	1.102	0.118-10.281	1.000^b^
Type of Fracture	NOF^c^ vs IT^d^	30 (46) vs 35 (54)	0.444	0.104-1.899	0.319^b^
Surgical Treatment	Yes vs No	23 (35) vs 42 (65)	1.263	0.317-5.030	0.733^b^
ASA^e^ Grade	III-V vs I-II	51 (78) vs 14 (22)	0.583	0.129-2.628	0.438^b^
Hypertension	Yes vs No	38 (58) vs 27 (42)	1.806	0.422-7.730	0.673^b^
Diabetes Mellitus	Yes vs No	12 (18) vs 53 (82)	0.444	0.051-3.889	0.673^b^
Inpatient Morbidity	Yes vs No	27(42) vs 38 (58)	1.500	0.388-5.797	0.729^b^

*Statistically significant (p<0.05); ^a^Confidence Interval; ^b^Fisher’s Exact Test; ^c^Neck of Femur; ^d^Intertrochanteric; ^e^American Society of Anaesthesiologists

Prediction of one-year mortality was also investigated using multivariate logistic regression which confirmed age to be a significant predictive factor for one-year mortality (OR 1.646; P=0.002). Nagelkerke’s R2 for this model was 0.303 suggesting that age alone accounted for 30.3% of the variability when predicting one-year mortality.

Prior to injury, 10 patients were already immobile and 55 patients were mobile. Amongst these 55, 37 underwent surgery and 18 non-operative management. A further eight patients (five from operative, and three from non-operative group) had passed away within six months before ambulatory status was achieved. Since it was not known whether they were eventually ambulated if not for early demise, they were also excluded from mobility analysis. Therefore about 28% of patients from the 65 studied were not able to have their mobility compared post injury.

We were able to show that 20 out of 32 patients (63%) of patients that underwent surgery were able to regain some form of ambulation (with aid or assistance) within the follow-up period. Only one out of 15 patients (7%) of conservatively managed patients were able to regain ambulation (p<0.001).

## Discussion

As life expectancy increases, we can expect more nonagenarians with hip fractures presenting to orthopaedics. Studies have shown that that advanced age and ASA scores are risks for mortality after hip fracture surgery^4,12-17^. Thus, the safety of operating on a nonagenarian with a hip fracture may be questioned. To address this, we have analysed the mobility, morbidity and mortality in nonagenarians with hip fractures with regards to both operative and non-operative management. There is also some relevant literature, discussed below, that specifically addresses hip arthroplasty^18-21^ and hip fractures^6,12,13,22-31^ in nonagenarians.

One of the main aims was to assess the inpatient morbidity in our patient group. The occurrence of morbidity at 41.5% was lower than other studies reporting rates between 52% and 78%^6,8,12^. Urinary tract infection was the most common morbidity in both surgically and conservatively managed patients.

An important determinant we found to significantly increase the chance of inpatient morbidity was having a higher ASA score (3 to 5). This finding is not surprising and emphasises the need for more careful monitoring of patients with multiple or significant co-morbidities.

Inpatient morbidity is important to prevent and reduce cost to patient and shorten their length of stay in hospital. Mortality outcomes are reported in most of the studies, but only a few studies have shown comparative results with a younger population of hip fractures at their institution.

Mehul Shah *et al*^30^ showed in an operated hip fracture population, nonagenarians were compared to those less than age of 90. Nonagenarians were more likely to have an ASA of 3 or 4, more likely to die within the hospital admission (10.6% vs 2.1%) and have a higher 1-year mortality (25% vs 10%).

Vochtelo *et al*^6^ studied hip fractures in Netherlands and found that nonagenarians were more likely to: have longer hospital stay; have anaemia on admission; require blood transfusion; suffer with delirium or cardiac complications. One-year mortality was 42.6% compared to 23.2% amongst younger patients aged 65-89.

Kadowaki *et al*^13^ published a series of hip fractures in a Japanese population. About a quarter of their surgically managed hip fracture population was over the age of 90. Compared to younger patients with a 1-year mortality of 10%, those over the age of 90 had a 26% mortality rate. They correlated mortality strongly with the loss of ability to walk after surgery.

Worldwide literature reports 1-year mortality rates in nonagenarians with hip fractures to be range from 25% to 46%^6,8,12,13,27,30^. With regards to results in our local Singaporean population, there are only two relevant studies evaluating the outcomes of hip fracture surgery in nonagenarians^8,32^. Ooi *et al*^8^ reported a 30% mortality rate at one year post hip fracture surgery. Tay *et al*^32^ showed a lower 1-year mortality rate of 12.1%. The latter study was published 10 years after the former, so the variation may be partly explained by evolution in healthcare approaches towards the elderly.

When looking at the general population of hip fractures, (not specifically nonagenarians) some studies have compared outcomes of surgery versus no-surgery. Averkieva *et al*^33^ studied 261 elderly patients with hip fractures that were evenly distributed between conservative and surgical management, it was evident that at 6 months, the mortality rate of the conservative group (32%) was far higher than the surgically managed group (6%).

Tay *et al*^7^ showed in a Singaporean population of 340 hip fractures (patients above 60 years of age), the overall mortality rate was an acceptable 14.4% at one year. The 33.5% of patients who were treated conservatively had a 30% 1-year mortality rate compared to the 6.6% 1-year mortality rate for those treated with surgery. Although the non-operative cohort was slightly older, there was no significant difference in ASA grade compared to the operated patient group, and ASA grade did not influence mortality in this study.

To our knowledge, the ‘surgery versus no-surgery’ comparison for mortality in nonagenarian hip fractures has seldom been reported. Ooi *et al*^8^ found within an overall 37% 1-year mortality rate, the breakdown was 30% (surgical) vs 46% (non-surgical). This difference, while appearing large, did not reach statistical significance.

On summarising the literature findings on mortality, the numerous studies presented above make two distinct arguments. Firstly, amongst surgically treated hip fractures, nonagenarians have higher mortality at one year compared to younger aged patients, and this ranges from 14-46% (with our study included). Secondly treating hip fractures non-surgically may result in a higher risk of death within one year, and this is not just the case for nonagenarians. Our own results reveal that advancing age was a factor that increases the mortality rate after hip fracture. Therefore, while nonagenarians are at higher risk of mortality, they should still be considered for surgery.

Our study reports a total mortality rate of 14.3% amongst surgically managed nonagenarians which is generally lower than most studies in the literature, and similar to another local study reported by Tay *et al*^32^.

We also found that there was no significant difference in 1-year mortality between our patients managed conservatively (17.4%) and surgically (14.3%). This is an important finding that contrasts with expectations and the aforementioned evidence. We suggest that while nonagenarians should certainly be considered for surgery, conservative management does not necessarily mean their days are numbered. With appropriate institutional or home care support, the risk of medical complications and death can be reduced.

With regard to mobility in nonagenarian populations, previous studies have shown 16%-57.3% of patients maintain mobility after hip fracture surgery^6,24,25,30^. Compared to conservative treatment, others have reported that surgery significantly increases the ability to ambulate independently^8^.

As with current literature, we found that 63% of previously ambulatory patients from the surgical group were able to ambulate to some degree after surgery. As expected, only 7% of conservatively managed patients (who were mobile pre-injury) could ambulate again. Surgery clearly meets its aim to restore function and ambulatory status, compared to non-operative management.

Our study has several limitations. This is an observational retrospective study and given there were only 42 operated cases and 23 non-operative cases, comparisons between these two groups has to be interpreted with caution. Mobility outcomes were more of an observation and multivariate analyses were not performed for several reasons. Firstly, there was significant loss of follow-up for this result (28%).

Secondly, we could only use a non-quantitative means of assessing post injury mobility. The NMS could be better measured pre-injury when taking a history from patients. However, activities such as leaving the house and shopping were tasks that a patient recovering from hip fracture may not have done due to other factors. Often these could be cultural influence where family discourage it after a patient has already had a significant fall. It could also be practical reasons such as if supervision/physical assistance is needed for ambulation. If supervision is not available for longer periods to enable community ambulation then patient will rarely explore the community. The value of the NMS tool in this age group is debatable. As the ability to leave the house and do shopping is not truly an indicator of mobility alone and can be influenced by other patient factors such as poor vision, or dementia.

Lastly, we tried to assess ambulatory status mobility at six months (or earlier if this was achieved earlier) but patients did not adhere to specific follow-up periods and some patients’ mobility may have been assessed closer to three months and some closer to one year. Therefore, this study does not provide answers as to when ambulatory status can be expected after injury.

## Conclusion

In summary this study demonstrates a few key findings within a nonagenarian hip fracture population. In our institution a considerable proportion of patients are treated conservatively. However, the main effect of conservative treatment is that of significant reduction in regaining mobility compared to operated patients. Mortality, however, was similar regardless of treatment option (17.4% conservative versus 14.3% operative). Our overall one-year mortality rates and inpatient complications in Singapore are lower than that suggested by other parts of the world. The most influential factor on mortality was increasing age at presentation and possibly obtaining an inpatient medical complication. Morbidity was more likely in patients with ASA 3-5. Surgery significantly improves the chances of post-operative mobility without increasing mortality risk, and should be advocated when possible, with careful attention to those with additional co-morbidities.
